# Disease severity of community-acquired pneumonia among children with medical complexity

**DOI:** 10.1002/ppul.26269

**Published:** 2022-12-20

**Authors:** Sriram Ramgopal, Jillian M. Cotter, Nidhya Navanandan, Lilliam Ambroggio, Todd A. Florin

**Affiliations:** 1 Division of Emergency Medicine, Ann & Robert H. Lurie Children’s Hospital of Chicago, Northwestern University Feinberg School of Medicine, Chicago, Illinois, USA; 2 Department of Pediatrics, Section of Pediatric Hospital Medicine, Children’s Hospital Colorado, University of Colorado, Aurora, Colorado, USA; 3 Department of Pediatrics, Section of Emergency Medicine, Children’s Hospital Colorado, University of Colorado, Aurora, Colorado, USA; 4 Department of Pediatrics, Sections of Emergency Medicine and Hospital Medicine, Children’s Hospital Colorado, University of Colorado, Aurora, Colorado, USA

To the editor,

The use of objective outcome measures that can be “standardized, measured, and compared” have been recognized as a key priority in the Infectious Diseases Society of America and Pediatric Infectious Disease Society pediatric community-acquired pneumonia (CAP) guideline.^1^ However, to date, most research on pediatric CAP has been limited to surrogate outcomes such as hospital length of stay or revisits, which lack granularity and objectivity.^2^

We previously demonstrated that a four-tiered ordinal outcome based on objective clinical outcomes (e.g., need for supplemental oxygen, or use of a thoracostomy tube) may help fill this research gap.^3^ In a recently published study that applied these objective outcomes to children without medical complexity, we identified that most (68%) children with CAP do not require hospitalization. In addition, we found that a substantial proportion of children that are hospitalized (20%) may have a potentially unnecessary admission.^4^ However, these criteria have not been applied to children with medical complexity. One prior study evaluating pneumonia outcomes among children with medical complexity noted that these children had longer lengths of stay, greater costs, and need for readmission compared to children with pneumonia but without medical complexity.^2^ No large study to date has evaluated the severity of disease in children with medical complexity using granular measures. We therefore evaluated the severity among children with medical complexity using this four-tiered objective ordinal measure.

We performed a multicenter cross-sectional study using data abstracted from 41 hospitals in the Pediatric Health Information System (PHIS), an administrative database that contains emergency department and inpatient data from geographically diverse US children’s hospitals. The investigation was designated as exempt by our Institutional Review Board. We included children, 90 days to 18 years of age, with a complex chronic condition (CCC) who presented to a participating hospital between January 1, 2016 to March 31, 2020, and with a primary diagnosis of pneumonia.^4^ We retained the first encounter per patient. CCCs were defined as medical conditions expected to last >12 months and require specialty care and/or hospitalization in a tertiary care center using encounter-level diagnoses and were identified using criteria described by Feudtner et al.^5^ Each International Classification of Disease, 10th revision (ICD-10) diagnosis can be grouped into 1 of 10 mutually exclusive diagnosis categories and additionally be classified as technology dependence, transplant, or both.

We classified encounters using a four-tiered ordinal severity measure: mild-discharged, mild-admitted, moderate, and severe (Supporting Information: Table). Mild-discharged patients were discharged from the ED without readmission within 7 days. Mild-admitted patients were admitted on the index visit or a revisit but did not meet any other criteria to fall into a higher tier during either encounter. Moderate disease severity was defined as a hospitalized patient who was given intravenous fluid, oxygen, broadening of antibiotics (from an aminopenicillin), had a complicated pneumonia, or who had presumed sepsis. Severe disease was classified as requiring the intensive care unit, positive-pressure ventilation, vasoactive infusion, chest drainage, ECMO, severe sepsis, or in-hospital mortality. We evaluated the demographics of disease severity among this cohort of children by medical complexity and reported differences in severity based on the type and number of CCCs (1, 2, or ≥3), and evaluated differences in presentation by hospital. Given that some children with medical complexity require chronic bilevel or continuous positive airway pressure and specific institution-based ICU requirements for these patients vary, we performed a sensitivity analysis in which we modified our criteria to remove the ICU admission criteria and limited the positive pressure ventilation criterion to only include those patients without use of positive pressure ventilation on any previous hospital encounter. We performed our analysis with R, version 4.1.2 (R Foundation for Statistical Computing).

Overall, 20,447 children with CAP and a CCC were identified from the data set (median age 4 years, IQR 2–9 years). Approximately half (56.2%) had a single CCC. Using the ordinal severity outcome, 3997 (19.5%) were classified as mild-discharged, 2338 (11.4%) as mild-admitted, 8651 (42.3%) had moderate disease, and 5461 (26.7%) had severe disease (Table 1). Among patients with moderate disease, intravenous fluids (43.8%) was the single most prevalent factor, followed by use of supplemental oxygen (17.2%). Among those with severe disease, ICU admission (21.3%) was the most prevalent factor, followed by positive pressure ventilation (19.1%). Ninety-seven children (0.4%) died in the hospital. More CCCs increased the likelihood of greater severity (Table 2). Among types of CCC, greater disease severity was identified among those with respiratory, neuromuscular, or metabolic comorbidities, and technology dependence. There was a substantial variation in disease severity in admitted children across hospitals. Among the included hospitals, the median % of patients with mild-admitted acuity was 11.2% (IQR 7.0%–21.4%), and the median % of patients with severe acuity was 30.1% (IQR 24.8%–35.4%) (Supporting Information: Figure). When using a modified severity measure in our sensitivity analysis, overall results were similar with 20.0% of children in mild-discharged, 12.1% mild-admitted, 45.0% moderate, and 22.8% with severe disease.

In this multicenter study, we evaluated disease severity among children with medical complexity with a diagnosis of CAP. The present study expands upon our work evaluating pneumonia severity in children without comorbidities, which demonstrated that 68.3% were classified as mild-discharged, 6.6% as mild-admitted, 20.6% as moderate, and 4.5% as severe when using the same criteria applied to children from the same hospitals from the same time.^4^ In contrast to children without CCC, severe outcomes occurred more frequently among patients with CCCs (26.7% vs. 4.5%). Our findings demonstrate the substantial burden of disease among medically complex children with CAP. By identifying clinically relevant outcomes that contribute to disease severity, we further expand upon prior work describing CAP in children with medical complexity.^2^

Our work among previously healthy children suggests the need for improved risk stratification efforts among children with CAP to avoid potentially unnecessary hospitalizations (i.e., the mild-admitted group, which represented 20% of the hospitalized cohort). In contrast, the present study demonstrates that fewer children with CCCs had potentially unnecessary admissions (14% of admitted patients). However, we identified substantial variability in pneumonia severity classification across hospitals suggesting that even in this higher-risk cohort, institutional practice patterns play a substantial role. Clinical pathways for the management of CAP in children should take our findings into consideration, particularly among those with higher risk comorbidities. These pathways may additionally serve to reduce unnecessary variability.

Children with CCC account for a substantial proportion of all pediatric mortality, and the challenge of caring for children with medical complexity is likely to increase over time. A longitudinal assessment of nationally representative survey data, for example, suggests that rates of CCC are increasing in complexity and frequency, underscoring the importance of better meeting the needs of these children.^6^ Our data, when combined with this literature, suggests that children with CCC who present with CAP warrant increased attention given their higher risk for severe disease. Children with medical complexity represent a diverse population with respect to their risk of disease severity with CAP, with children with certain conditions having a higher risk of severe illness. As such, predictive models, and clinical practice guidelines for children with CCC would potentially aid clinicians’ decision-making with respect to diagnostic testing, empiric antimicrobial utilization, and hospitalization. It is important that such guidelines consider CCC type given the heterogeneity of this population.

Our findings are subject to limitations. We used ICD-10 codes to identify patients with pneumonia, which may result in mis-classification. We were not able to distinguish between bolus and continuous intravenous fluids. Additionally, PHIS lacks medical data (e.g., vital sign, physical examination, and test results) and social information that may be important when considering the need for hospitalization. However, there remains utility in applying an objective measure to severity that does not consider these criteria (some of which are more subjective) and which can readily be applied to a large data set. Despite these limitations, the findings from this study provide useful data on disease severity among children with CAP and medical complexity.

Using a previously developed composite outcome applied to children with CAP and CCCs, we identified that children with CCCs presenting to the ED had a higher proportion with moderate and severe disease severity, with certain subtypes of CCCs having the greatest risk. Using this system, 19.5% had mild, 11.4% had mild-admitted, 42.3% had moderate, and 26.7% had severe disease. These findings suggest that a low proportion of children with CCCs have potentially unnecessary admissions. There was extensive variability in the severity distribution among the included hospitals. Predictive models constructed to predict disease severity in this population should carefully consider the extent and type of a child’s medical complexity.

## Supplementary Material

supp fig

supp table

## Figures and Tables

**TABLE 1:** Demographics, diagnostic testing, and use of antibiotics among children with community-acquired pneumonia and medical complexity

	Overall	Mild-discharged	Mild-admitted	Moderate	Severe
	*N* = 20,447	*N* = 3997	*N* = 2338	*N* = 8651	*N* = 5461
Admission age	4 [2–9]	5 [2–10]	4 [1–8]	4 [2–9]	4 [2–10]
Male sex	11,244 (55.0)	2268 (56.7)	1331 (56.9)	4685 (54.2)	2960 (54.2)
Payor status					
Other	1000 (4.9)	277 (6.9)	138 (5.9)	375 (4.3)	210 (3.8)
Private	6889 (33.7)	1173 (29.3)	834 (35.7)	3013 (34.8)	1869 (34.2)
Public	12,558 (61.4)	2547 (63.7)	1366 (58.4)	5263 (60.8)	3382 (61.9)
Diagnostic testing					
Chest radiograph	18,368 (89.8)	3573 (89.4)	1960 (83.8)	7631 (88.2)	5204 (95.3)
Chest computed tomography	815 (4.0)	13 (0.3)	43 (1.8)	273 (3.2)	486 (8.9)
Chest ultrasound	889 (4.3)	5 (0.1)	23 (1.0)	243 (2.8)	618 (11.3)
Blood culture	10,972 (53.7)	1024 (25.6)	1114 (47.6)	5054 (58.4)	3780 (69.2)
Complete blood count	14,503 (70.9)	1366 (34.2)	1589 (68.0)	6738 (77.9)	4810 (88.1)
C-reactive protein	7064 (34.5)	522 (13.1)	633 (27.1)	3194 (36.9)	2715 (49.7)
Procalcitonin^a^	2008 (9.8)	134 (3.4)	92 (3.9)	845 (9.8)	937 (17.2)
Respiratory viral panel	6123 (29.9)	709 (17.7)	645 (27.6)	2681 (31.0)	2088 (38.2)
Respiratory culture	1582 (7.7)	105 (2.6)	69 (3.0)	342 (4.0)	1066 (19.5)
Antibiotics during hospital encounter	17,190 (84.1)	1926 (48.2)	2089 (89.3)	8064 (93.2)	5111 (93.6)
Length of stay in days, median	2 [1–5]	1 [1–1]	2 [1–3]	3 [2–4]	6 [3–11]
Standardized cost, median	7390 [3000–16,900]	654 [404–1210]	5200 [3290–8140]	7690 [5100–13,400]	22,500 [12,100–43,000]

*Note*: Numbers in parentheses represent column %. Numbers in brackets represent interquartile ranges.

a Procalcitonin was assessed using orders for calcitonin (which included procalcitonin).

**TABLE 2:** Distribution of pneumonia severity by number and type of complex chronic condition (CCC)

	Mild-discharged	Mild-admitted	Moderate	Severe
	*N* = 3997	*N* = 2338	*N* = 8651	*N* = 5461
Number of CCCs				
1	3155 (27.5)	1459 (12.7)	4979 (43.3)	1893 (16.5)
2	704 (13.7)	595 (11.6)	2328 (45.4)	1501 (29.3)
≥3	138 (3.6)	284 (7.4)	1344 (35.1)	2067 (53.9)
Type of CCC				
Respiratory	463 (12.3)	305 (8.1)	1226 (32.7)	1760 (46.9)
Neuromuscular	633 (13.0)	395 (8.1)	1835 (37.5)	2024 (41.4)
Metabolic	179 (8.6)	154 (7.4)	891 (43)	848 (40.9)
Technology dependence	1153 (14.1)	789 (9.6)	3086 (37.6)	3170 (38.7)
Gastrointestinal	938 (12.6)	740 (9.9)	2937 (39.3)	2857 (38.2)
Cardiovascular	710 (16.4)	509 (11.8)	1634 (37.7)	1476 (34.1)
Transplant	3 (4.4)	6 (8.8)	36 (52.9)	23 (33.8)
Neonatal	151 (8.3)	213 (11.7)	851 (46.9)	598 (33.0)
Renal	170 (12.6)	143 (10.6)	593 (44)	443 (32.8)
Congenital/genetic	935 (18.3)	515 (10.1)	2214 (43.4)	1434 (28.1)
Malignancy	337 (21.6)	185 (11.9)	729 (46.8)	307 (19.7)
Hematologic/immune	495 (19.0)	410 (15.7)	1207 (46.2)	499 (19.1)

*Note*: Numbers in parentheses represent row %.
