# Supplementary figures and images for: Disease severity of community-acquired pneumonia among children with medical complexity
Source: Pediatr Pulmonol. Author manuscript; Available in PMC 2024 Aug 9. (PMC11311197; doi:10.1002/ppul.26269)

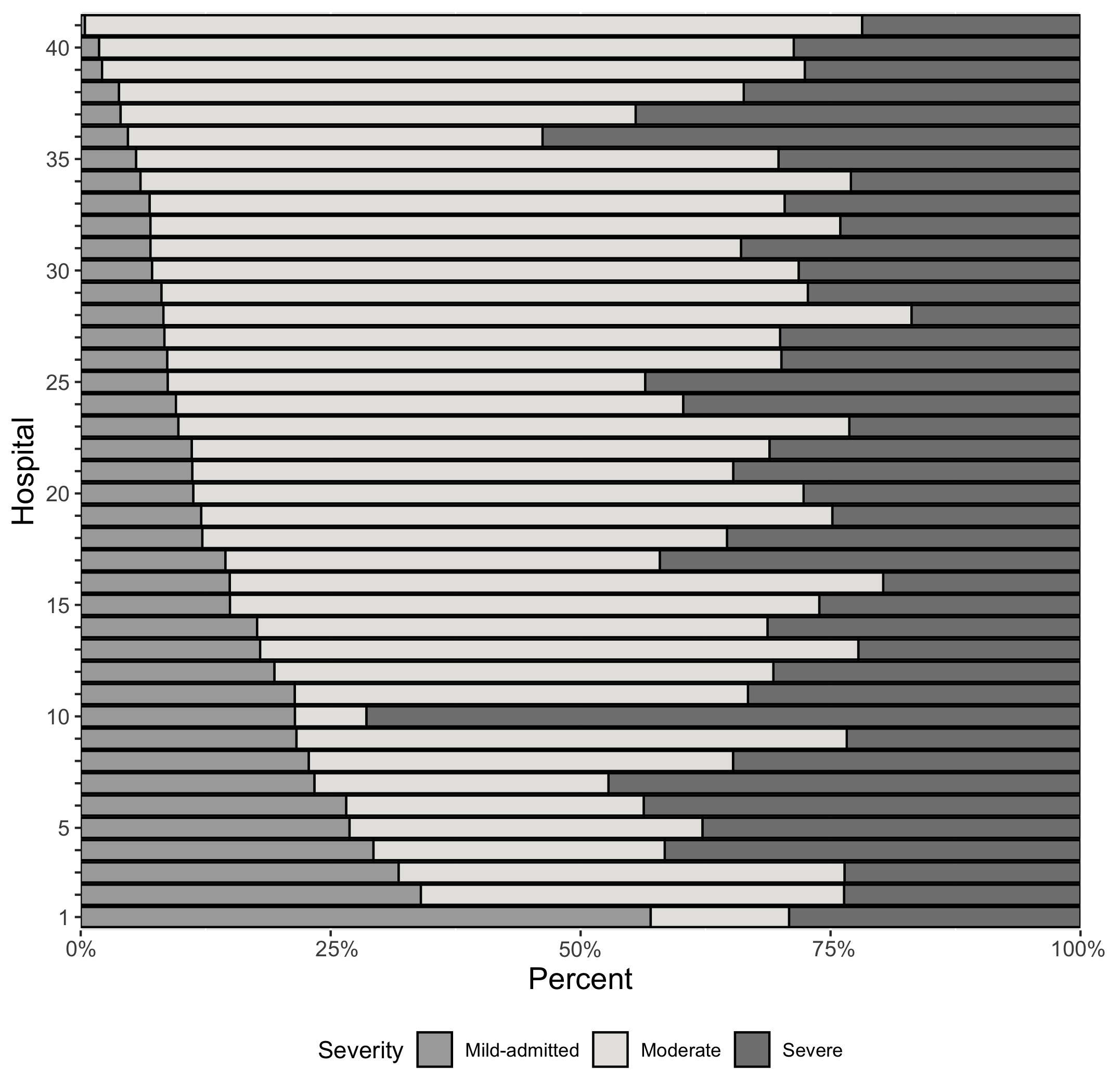

Supplement: supp fig [file NIHMS2014706-supplement-supp_fig.tif]
