# Supplementary material for: Disease severity of community-acquired pneumonia among children with medical complexity
Source: Pediatr Pulmonol. Author manuscript; Available in PMC 2024 Aug 9. (PMC11311197; doi:10.1002/ppul.26269)
Supplement: supp table [file NIHMS2014706-supplement-supp_table.docx]

**Supplementary Table.** Four tier severity outcome

| **Criteria** | **Criteria Adaptation to PHIS** | **Modified Criteria for Sensitivity Analysis** |
| --- | --- | --- |
| **Mild-Discharged** | No admission, AND  No revisit leading to readmission <7 days from index visit | No admission, AND  No revisit leading to readmission <7 days from index visit |
| **Mild-Admitted** | Admitted from index visit OR admitted from revisit  AND  No additional criteria met from the moderate or severe groups | Admitted from index visit OR admitted from revisit  AND  No additional criteria met from the moderate or severe groups |
|  | | |
| **Moderate** | Hospitalized AND provision of any IV fluids  *and/or*  Hospitalized AND provision of supplemental oxygen  *and/or*  Hospitalized with broadening of antibiotics from an aminopenicillin  *and/or*  Hospitalized, AND presence of an additional diagnosis code for that encounter  *and/or*  Hospitalized, AND presence of a systemic inflammatory response syndrome or sepsis diagnosis code | Hospitalized AND provision of any IV fluids  *and/or*  Hospitalized AND provision of supplemental oxygen  *and/or*  Hospitalized with broadening of antibiotics from an aminopenicillin  *and/or*  Hospitalized, AND presence of an additional diagnosis code for that encounter  *and/or*  Hospitalized, AND presence of a systemic inflammatory response syndrome or sepsis diagnosis code |
|  | | |
| **Severe** | ICU admission  *and/or*  Use of positive pressure ventilation (using a dedicated flag, a procedure code for endotracheal intubation, provision of CPAP or BiPAP, or an ICD-10 code for Z99.11 (Dependence on ventilator status)  *and/or*  Vasoactive infusion with provision of parental (epinephrine, norepinephrine, dopamine, milrinone, dobutamine, phenylephrine, or ephedrine)  *and/or*  Performance of any drainage procedure of the chest, lung, pleura, bronchus or pericardium  *and/or*  Use of ECMO  *and/or*  Hospitalized, and presence of a severe sepsis diagnosis code  *and/or*  In-hospital mortality | Use of **new-onset** positive pressure ventilation (using a dedicated flag, a procedure code for endotracheal intubation, provision of CPAP or BiPAP, or an ICD-10 code for Z99.11/ICD-9 code for V46.11 (Dependence on ventilator status) **relative to previous use**  *and/or*  Vasoactive infusion with provision of parental (epinephrine, norepinephrine, dopamine, milrinone, dobutamine, phenylephrine, or ephedrine)  *and/or*  Performance of any drainage procedure of the chest, lung, pleura, bronchus or pericardium  *and/or*  Use of ECMO  *and/or*  Hospitalized, and presence of a severe sepsis diagnosis code  *and/or*  In-hospital mortality |

ICD, International classification of disease; ECMO, extracorporeal membrane oxygenation; ICU, intensive care unit; CPAP, continuous positive airway pressure; BiPAP, bilevel positive airway pressure
